# Prospective Cohort Study on the Effect of an Intervention to Reduce Household Air Pollution Among Sudanese Women and Children

**DOI:** 10.5696/2156-9614-11.31.210905

**Published:** 2021-08-17

**Authors:** Alawia K Suliman, Maysoon M. Saleh, Kristin Sznajder, Tonya S. King, W. Stuart Warren

**Affiliations:** 1 Department of Pediatrics, Penn State College of Medicine, Hershey, Pennsylvania; 2 Department of Pediatrics, Red Sea University, Port Sudan, Sudan; 3 Department of Public Health Sciences, Penn State College of Medicine, Hershey, Pennsylvania

**Keywords:** biomass fuels, household air pollution, Sudan, intervention study, women's health, children's health

## Abstract

**Background.:**

Exposure to household air pollution through the burning of biomass fuels is a global health concern and can lead to negative health outcomes such as asthma and lung disease.

**Objectives.:**

The goal of this four-year study was to determine whether an intervention to reduce household air pollution (HAP) which included health education and a new well-ventilated cooking location would reduce exposure to HAP, lower carbon monoxide (CO) levels and improve the health of women and children in Port Sudan, Sudan.

**Methods.:**

In 2016, 115 women of low socioeconomic status and their children were invited to participate in the study at two women's centers. One hundred and eleven women consented to participate and were divided into study and control groups on the basis of home ownership. Women who owned their homes learned about the adverse effects of HAP and a well-ventilated outside cooking location (*rakoobah*) was provided. Control women did not receive HAP education or a *rakoobah*. Questionnaires were used to assess the effect of education and a new well-ventilated cooking location for a group of Sudanese women who cook with biomass fuels. CO-oximetry was performed. Each year from 2017–2019, the questionnaires and CO-oximetry were repeated.

**Results.:**

Sixty-five women and 33 children were assigned to the study group and 46 women and 19 children were assigned to the control group in 2016. Women were enrolled in 2016 with CO levels of 17.8% and 17.4%, respectively. One year later some of the study group women had lower CO levels and others higher, while the CO levels of the controls were stable. An intensive HAP education program was started for the study group women. By 2019, the last study year, the CO levels of both the study and control group women had dropped to normal levels of 2.9% and 3.1%, respectively. Control group women may have benefited from the HAP education and modeled behavior of those in the study group.

**Conclusions.:**

The health impact of the change in cooking location was unclear, yet both groups reported fewer health care visits in 2019. Education and an outside cooking location resulted in lower CO levels of Sudanese women and children.

**Participant Consent.:**

Obtained

**Ethics Approval.:**

The study was approved by the Penn State Milton S. Hershey Medical Center Institutional Review Board and the Ethics Committee of the Red Sea University Faculty of Medicine located in Port Sudan, Sudan.

**Competing Interests.:**

The authors declare no competing financial interests.

## Introduction

Energy poverty represents a serious challenge in sub-Saharan Africa. Only 12% of Africans use cook stoves that run on fuels such as liquefied petroleum, electricity or renewable fuels such as biogas, ethanol or solar energy.^1^ Thus, the vast majority of households rely on biomass fuels for cooking which traditionally occurs in small poorly ventilated dwellings and produces the smoky air characteristic of household air pollution (HAP). Evidence from the World Bank 2014 report shows that approximately 600 000 Africans die annually and many more suffer from chronic illnesses due to HAP.^1^

Monitoring of indoor air pollution has been carried out primarily by measuring indirect exposure in dwellings and on the clothes of residents.^2^ These techniques measure the primary products of partial biomass fuel combustion: carbon monoxide (CO), nitrogen dioxide (NO_2_), sulfur dioxide (SO_2_), and particulate matter (PM)^2^ and are associated with variations of exposure within households and sub-groups. Carbon monoxide is absorbed into the body after inhalation and blood levels can be measured. Health-related effects similar to those reported with HAP exposure have been reported with CO poisoning including symptoms involving the heart,^3^ central nervous system,^4^ and placenta.^5^

In sub-Saharan Africa, where the majority of the population cook indoors with biomass fuels, global efforts continue to develop improved stoves which may reduce HAP exposure.^6^ Women and younger children have the highest exposure to HAP due to the disproportionate amount of time spent cooking compared with men or older children.^7^–^10^ Behavioral studies have looked at cooking practices but have not looked at the importance of cooking location nor measured the CO levels associated with improved cookstoves or changes in cooking location.^11^

In 2015, Suliman and colleagues carried out a study in Port Sudan, Sudan which assessed the CO levels of women and children living and cooking in small poorly ventilated dwellings with biomass fuels or gas using CO-oximetry.^12^ Data points included CO levels and several health effects of CO poisoning. Women who cooked with biomass fuels had mean CO levels of 18%. The women who cooked with gas had mean CO levels of 12%. Both groups had symptoms of CO poisoning. The current study was a natural follow up, and an effort to educate women about the effects of HAP and to provide them with a cooking location with lower HAP exposure and an opportunity to decrease the symptoms of CO poisoning. The study hypothesis was that regular cooking in a well-ventilated cooking site would result in a 50% drop in CO levels, measured using CO-oximetry, among study participants and some abatement of their CO poisoning symptoms.

**Abbreviations**
*HAP*: Household air pollution

The goal of this four-year study was to determine whether an intervention to reduce household air pollution (HAP) which included health education and a new well-ventilated cooking location would reduce exposure to HAP, lower carbon monoxide (CO) levels and improve the health of women and children in Port Sudan, Sudan.

## Methods

Port Sudan, a large city in eastern Sudan, was chosen for the present study due to the presence of a large population of lower socioeconomic status women with children between 1–5 years of age living in small poorly ventilated dwellings who cook with biomass fuels. These women congregate in government-sponsored women's centers where they are taught to read and write. Members of the faculty of the Redsea University Faculty of Medicine were interested in the study and had access to the target population. A previous study which enrolled this Port Sudan population had been successfully carried out.^12^

A Sudanese research team was recruited to carry out the present study, which included a field director, a study director, three female physicians, and six women's center counselors. Information sheets were developed for the Sudanese research team and study participants to explain the effects of HAP, the study purpose, the study consent form, and the inclusion of children under 5 years of age. Because study participants were illiterate or semiliterate, a verbal consent form was composed according to the guidelines of the Penn State Milton S. Hershey Medical Center Institutional Review Board. Permission to include children in the present study was part of the verbal consent form.

Questionnaires for women and children were developed to obtain history of HAP exposure due to a variety of factors such as cooking fuels, tobacco use, incense use, and dwelling size *(Supplemental Material).* A second questionnaire was developed to obtain a history of potential health effects of HAP exposure *(Supplemental Material).* All documents were translated into Arabic and the accuracy of the translations were verified by third parties not involved in the study. CO-oximetry was performed on mothers and children. The Wong-Baker pediatric pain scale was used to assess headache severity.

### Ethics approval

The study was approved by the Penn State Milton S. Hershey Medical Center Institutional Review Board and the Ethics Committee of the Red Sea University Faculty of Medicine located in Port Sudan, Sudan.

### Participant enrollment

The present study was conducted over a four-year period from 2016–2019. Each year prior to starting/restarting the study, the health-related effects of HAP exposure, the consent process, the questionnaires and CO-oximeter usage were reviewed with the team. Counselors at the women's centers were initially trained to recruit potential subjects and each subsequent year were asked to gather previously-enrolled subjects for follow up evaluation as well as to evaluate the new cooking location at their weekly visits. Approximately 50–60 local women come regularly to learn to read and write at each of the two women's centers chosen for the study. All potential subjects recruited by the counselors were offered the opportunity to participate in the study. The research team reviewed the educational materials and consent form, which included parental consent for enrollment of children five years of age and younger. Of the 115 women who were recruited to participate in the study, 111 gave consent and were enrolled.

Subjects who owned their own home were recruited to the experimental group and subjects who did not own their home were recruited to the control group. Subjects were divided in this way because the construction of a well-ventilated cooking location was only possible if the property was owned by the woman or her family. However, this division could have introduced bias if women and their children who own their own homes were of a higher socio-economic status or higher education level and were at a lower risk of CO exposure compared with women who did not own their own homes. Following the consent process, demographic and subjective data on dwelling size, cooking fuels and health information were collected for the women and children in the present study. Carbon monoxide levels were measured in duplicate for both adults and children. Identical procedures were carried out by the research team in 2017, 2018, and 2019.

A well-ventilated outside cooking location [6 ft × 7 ft × 6 ft] without walls, a cooking site in one corner and a corrugated iron roof (*rakoobah*) was built for each subject who owned her own home and these women received a short discussion about the health effects of smoke-filled air associated with indoor cooking. Counselors made weekly visits to each *rakoobah* recipient to ensure regular use of the new cooking location. The women assigned to the control group did not own their home, received no *rakoobah* and no discussion about the health effects of smoky air.

In 2017, when the results of the first year of study became available, a more formal HAP education program was started for the *rakoobah* recipients and counselors. In bi-monthly meetings at the women's centers, the study director reviewed the negative health effects of breathing smoky air and the symptoms of chronic CO poisoning. The counselors continued their weekly home visits to reinforce and encourage regular *rakoobah* use. This process started in August of 2017 and continued into January 2018. The education programs at the women's centers continued throughout 2018 and concluded with the last study visit in February 2019.

### Equipment

Three Masimo Rad-57 CO-oximeters together with pediatric and adult sensors were provided by the Masimo Corporation, Irvine, California. The Masimo CO-oximeter is a non-invasive monitoring platform which uses eight wavelengths of light to measure oxygen and carbon monoxide saturation. The Masimo Corporation reports a standard deviation for accuracy of the CO-oximeter is +/− 3%,^13^ while Zaouter and Zavorsky^14^ report readings of +4% to −6% when the true COHb% is in the 10–14% range. The CO-oximeters and sensors were tested each year prior to starting the study and daily during the study. The CO determinations for the three instruments were consistent with values ranging from 1–3% when performed on the investigators. Normal CO values as measured by the CO-oximeter for individuals with no risk factors, such as tobacco use, are up to 5%. Levels of 10% or more are considered to be distinctly abnormal and may be associated with symptoms of CO poisoning.^15^

### Statistical analysis

Frequencies and percentages of potential sources of HAP exposure were reported and compared between the study and control groups using Chi-square and Fisher's exact tests. Mean CO levels and headache pain severity scores were estimated and compared between the study and control groups in repeated measures analysis of covariance models with the factor for year nested within groups. Results are reported in terms of model-estimated means, 95% confidence intervals and p-values. Frequency of headache, fatigue, weakness and dyspnea were compared between the study and control groups in repeated measures logistic regression models with the factor for year nested within groups. Results are reported in terms of annual percentages with p-values for trend estimated from the models. Cross-sectional comparisons from additional data collected in 2020 were performed using Fisher's exact tests. Significance was defined as p<0.05 and analyses were performed using SAS version 9.4 statistical software.

## Results

Sixty-five study group women who received a *rakoobah* plus some HAP education and their children (n=33) and 46 control women who did not receive a *rakoobah* nor HAP education and their children (n=19) were enrolled in 2016. Women were between the ages of 18–50 and the average years of education for the control group was 2.68, while the average number of years of education for the study group was 7.27. The study group included 16 male children (48.5%) and the control group included 10 male children (52.6%). The average age of children in both the study and control groups was 2.4 years. Factors potentially affecting levels of HAP exposure are listed in Table 1.

**Table 1:** Potential Sources of Household Air Pollution Exposure

	**Study (n=65)**		**Control (n=46)**		**P-value**

	**No of subjects**	**Percentage**	**No of subjects**	**Percentage**	
*Rooms in dwelling*					
One	50	76.9	29	63.0	0.28
Two	12	18.5	14	30.4	
Three	3	4.6	3	6.5	
*Cooking fuels*					
Charcoal/wood	65	100.0	46	100.0	—
Dung	4	6.2	2	4.4	0.68
Crop residues	42	64.6	31	67.4	0.76
Plastic	50	76.9	32	69.6	0.38
*Household lighting*					
Wood	2	3.3	0	0.0	0.50
Kerosene	9	15.0	11	23.9	0.25
Gas	0	0.0	0	0.0	—
Flashlight	50	83.3	35	76.1	0.35
*Other*					
Tobacco	10	15.4	6	13.0	0.73
Incense	63	96.9	45	97.8	0.77
Dukhon*	35	53.8	23	50.0	0.69

^*^A *dukhon* is a traditional Sudanese treatment akin to a sauna and tanning bed combined consisting of a small room and burning of scented wood.

**Figure 1:**
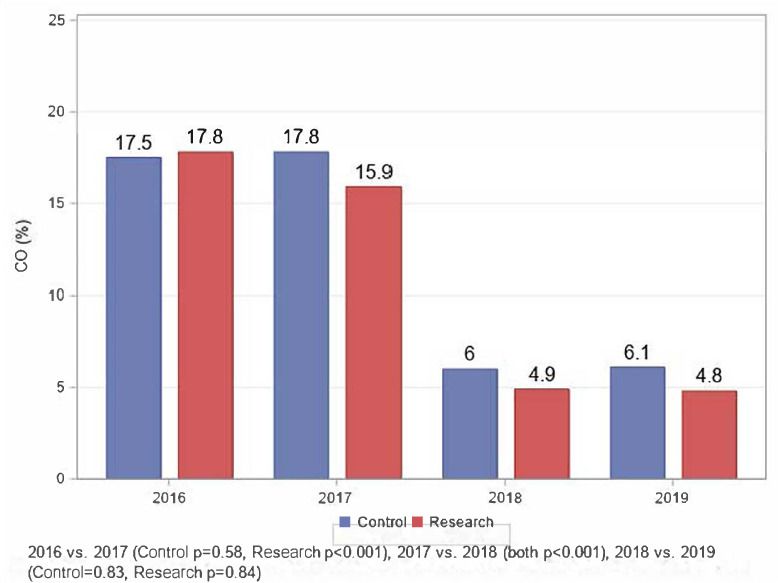
Carbon monoxide (CO) levels across study groups from 2016–2019

The course of the mean CO levels among women in the study and control groups is presented in Figure 1. At enrollment, the study group women had mean CO levels of 17.8% (95% CI 17.1–18.5), while the controls had mean CO levels of 17.5% (95% CI 16.7–18.3). One year later, 51 study group women and 31 controls were available for reevaluation. The CO levels in the study group dropped to a mean of 15.9% (95% CI 15.1–16.6), while the control group mean levels remained stable. Careful questioning of the study group women revealed that eleven women did not use their *rakoobah* at all and their mean CO levels were 19.2%. At the same time, the mean CO levels of the 40 compliant or semi-compliant participants were 14.8%. After 24 weeks of formal HAP education (2017–2018) by the study director, the mean CO levels of the study group dropped to 4.9% (95% CI 4.2–5.6) and the controls to 6.0% (95% CI 5.1–6.9). One year later, in 2019, the mean CO levels remained steady for both groups at 4.8% (95% CI 4.1–5.5) and 6.1% (95% CI 5.1–7.1), respectively.

**Figure 2:**
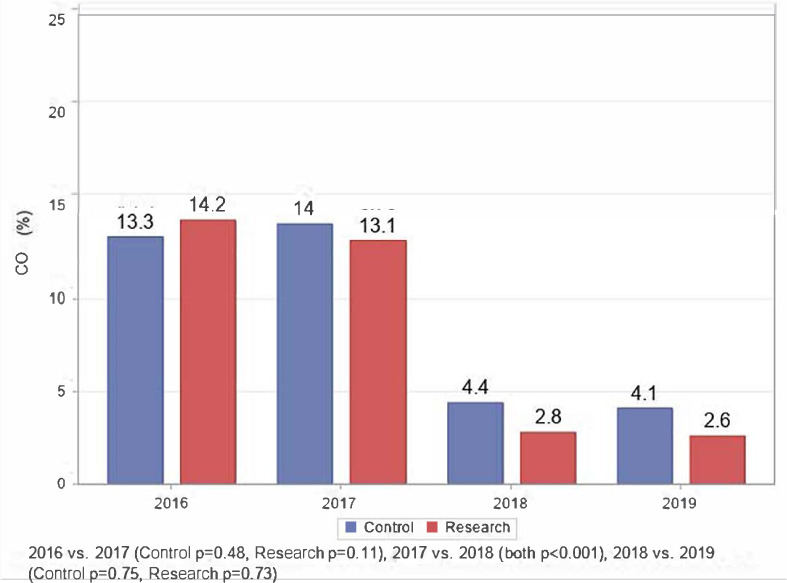
Carbon monoxide (CO) level in children across study groups from 2016–2019

Thirty-three children of mothers in the study group and 19 children of control mothers were enrolled. The children's mean CO levels at enrollment reflected their mothers' levels in the study and control groups: 14.2% (95% CI 13.3–15.2) and 13.3% (95% CI 12.0–14.6), respectively. As with the study mothers, the children's mean CO levels dropped slightly in 2017 while in the children of control mothers mean CO level increased slightly to 14.0% (95% CI 12.6–15.3). In 2018 and 2019 the drop in mean CO levels of the children mirrored the change in their mothers' levels, as shown in Figure 2.

Headaches were initially reported by almost all study and control women, 88% and 89%, respectively. Over the three study years, reports of headache decreased among study subjects (trend test p=0.004), but did not decrease among controls (trend test p=0.54). Reports of headache severity were measured using the Wong-Baker pediatric pain scale where subjects choose a picture reflecting the severity of their pain. The pain scale levels remained stable within each group although the controls consistently reported more severe pain with a mean score of 4.5 (95%CI 3.8–5.2) in 2019 compared with 2.4 (95%CI 1.8–3.0) among study subjects in the same year (p<0.001).

At enrollment, less than half of the study women reported dyspnea (45%), while only 33% of control women recognized this symptom. Over the three study years, 43% of the study group who initially reported dyspnea reported improvement in their dyspnea, while only 6% of the controls reported improvement. Even fewer study women at enrollment reported fatigue (40%) and weakness (25%), while fewer still of the control women reported fatigue (33%) and weakness (20%). Reports of improvement in these symptoms were inconsistent and varied without explanation from year to year. In 2019, 90% of the study women reported fewer visits for health care versus 76% of the controls (Fisher's exact p=0.13). In a brief 2020 follow up study all study women reported they were cooking in the *rakoobah*, while 19% of the controls reported they were not cooking outside. In an effort to better understand some of the differences between the control and study groups, information concerning years of schooling was collected. Sixty-seven percent of study women either reported not attending school or attending up to sixth grade (average 3.5 years) while 90% of control women reported no school attendance or attending up to sixth grade (average 1 year) (Fisher's exact p=0.08).

## Discussion

The hypothesis prior to the start of the study was that a well-ventilated cooking location would be associated with approximately 50% drops in the mean CO level and improvement in the symptoms of CO poisoning among study group women. In the first year of the study, 2016/2017, the team determined that for women with low socioeconomic status and who were unable to read or write, a change in cooking location and minimal education about HAP was not sufficient and the intervention required a much more robust educational component. In 2016–2017, 22% of the study group women failed to use their *rakoobah* and the others cooked in the *rakoobah* only sporadically. When the new more intensive education component was introduced, the study women started to use their *rakoobah* regularly and their mean CO levels dropped significantly.

Several mechanisms may explain why mean CO levels of the control women unexpectedly dropped in 2018. The control women may have participated in some of the bi-monthly education sessions carried out at the women's centers or the control women may have noticed improvement in the health of the women in the study group. This theory is substantiated by reports to the investigators by control women who noted that the women who cooked in the *rakoobah* appeared to feel better so they too began to cook outside and their CO levels also dropped.

The mean CO levels of the children in the study group reflected their mothers' levels. A small drop in CO levels in 2017 was followed by a large drop in 2018 which remained stable in 2019, while the control children, like their mothers, had a slight rise in 2017 followed by a big drop in 2018 and a stable 2019 level. This is likely because children spend a lot of their time held by or near their mothers. Therefore, interventions to reduce exposure to biomass fuels will usually improve both mothers' and children's CO levels.

Carbon monoxide, a colorless odorless gas, is excreted almost entirely in exhaled air. At rest, the elimination half-life of blood CO is approximately 320 minutes for a subject inhaling 21% oxygen or room air at normal atmospheric pressure.^16^ The half-life may increase with age and decrease with physical activity. Initially, elimination is rapid but then becomes slower when the level of carboxyhemoglobin drops. In this study the study and control subjects were breathing a mixture of smoky and fresh air, thus the elimination half-life must have been considerably longer. However, 24 weeks, the period of intensive HAP education, provided between late 2017 and early 2018, was sufficient to decrease study subjects' mean CO levels (measured by CO-oximetry) by 69% (15.9% to 4.9%) and the controls by 66% (17.8% to 6.0%); the study children by 79% (13.1% to 2.8%) and the control children by 69% (14.0% to 4.4%). The mean levels continued to drop during 2018 so that in early 2019 the levels were at normal physiologic levels of less than 5%.

Incomplete combustion of biomass fuels results in several pollutants: CO, SO_2_, NO_2_ and PM of various sizes. Carbon monoxide is the only product of incomplete biomass combustion which can be measured in the body. Exposure to CO can lead to a variety of lesions in the central nervous system which in turn can lead to reports of headache.^16^ The use of plastic, primarily bags, to start cooking fires is a different source of HAP and results in the release of toxic gasses such as dioxins, furans, polychlorinated biphenyl (PCB), and CO. Inhalation of these gasses aggravates chronic respiratory disease, may cause headache, and may affect the central nervous system.^16^ It is unclear how long the neurologic/headache recovery period is for chronic CO poisoning, but Betterman reported that neurologic outcome is highly variable with acute CO poisoning.^18^ Hampson reported that 35% of patients with acute CO poisoning had headaches after one year.^19^ There are no long term studies which report the neurologic outcome in individuals with chronic CO poisoning due to HAP nor due to long term dioxin/furan exposure which would help us to understand the study results.

It is unclear why women in the control group reported more headaches and greater severity of headaches throughout the study when the course of their mean CO levels paralleled the study group women and their exposure experience appeared to be similar. However, the women chosen to be controls were different in several respects. Not only did they not own their houses but they reported more visits for perceived health problems and fewer years of schooling for an average of 2.68 years compared with 7.27 years for the study subjects. As a result they may not have understood the questions, may have had more health problems overall, or have exaggerated their symptoms in an effort to get more help from the investigators or counselors. The decreased number of clinic visits for the study group could have been due to decreased symptoms related to CO exposure including cough, dyspnea, and fatigue; or perhaps a confounding effect of increased health education from the intervention.

Measuring a single pollutant may not be an adequate surrogate for other pollutants. Carter *et al.* reported that exposure to CO is not a reliable measure of exposure to particulate matter (PM_2.5_)^20^ Studies have not been performed correlating PM_2.5_ exposure with CO levels as measured by the CO-oximeter. Yet, the particulate matter in the smoky air of HAP is the major cause of respiratory disease in adults and children and there is strong evidence linking HAP exposure to chronic obstructive pulmonary disease and chronic bronchitis.^20^,^21^,^22^ In the present study, exposure to smoky air due to cooking was decreased but not eliminated, the levels of ambient air pollution were unchanged, and the use of tobacco, incense, and plastic continued. Thus, subjects and controls continued to inhale smaller amounts of the same pollutants producing persistent respiratory symptoms, albeit improved in some cases. Reports of weakness and fatigue were inconsistent over the study period among both study and control groups. Perhaps these women did not recognize these symptoms or considered this to be their normal state. An alternative explanation is that in this population of women there are other commonly found diagnoses which are associated with dyspnea, weakness and fatigue such as chronic lung disease, anemia, malnutrition and tuberculosis.

A key limitation was the assignment of women into the study and control groups. Women and their children in the present study were assigned to the study group if they owned their own home and to the control group if they did not own their own home. It is possible that this group assignment introduced bias if women in these two groups were different in their level of exposure to CO. However, all women in both the study and control groups cooked with three stone fires using biomass fuels. Another limitation of this study is the small number of study and control subjects enrolled and the failure of the investigators to factor in the nomadic nature of this population. As a result, 18–22% of the study group and 24–33% of the controls were not available for follow up in any given year. The study team questioned the counselors about the absentees and found two of the study subjects had died, three were seeking medical help, and two had very heavy henna changes on their hands which prevented CO-oximetry. An additional seven women in the study group were thought to be “traveling.” Similarly, many of the absent control women were thought to be “traveling.” At this season of the year both groups might have traveled to plant their crops. However, after year one (2017), the total number of subjects in each group was stable. A third limitation was the failure to measure CO levels at a greater number of time points which would have provided more data points and helped to better understand changes in CO levels over 3 years. A fourth study limitation was failure to consider and collect more data concerning the effects of incineration of other substances such as garbage, plastic and other potentially toxic products on ambient air pollution. The combustion of these products may have had a significant effect on the symptoms which we were trying to measure and explained the fact that in spite of achieving CO levels in a normal physiologic range, the symptoms of CO poisoning did not improve significantly. Further, data on how homes were heated and the duration of exposure to heating was not collected. In this area, many households use wood and charcoal to heat their homes.

## Conclusions

The present study demonstrates that an effective education program can result in major changes in cooking behavior, with changes in behavior lasting for more than one year. Changes in cooking behavior/location were associated with lower CO levels. There was a ripple effect among control women who began to cook outside when they realized the effects of the education program and recognized that women who were cooking in the *rakoobah* were experiencing health improvements. The results further suggest that a similar education program might be effective in reducing CO levels for a similar group of women and children where the goal is to change cooking behavior.
